# A portable thermal ablation device for cervical cancer prevention in a screen-and-treat setting: a randomized, noninferiority trial

**DOI:** 10.1038/s41591-024-03080-w

**Published:** 2024-06-25

**Authors:** Partha Basu, Mulindi Mwanahamuntu, Leeya F. Pinder, Richard Muwonge, Eric Lucas, Namakau Nyambe, Samson Chisele, Aaron Lunda Shibemba, Catherine Sauvaget, Rengaswamy Sankaranarayanan, Walter Prendiville, Groesbeck P. Parham

**Affiliations:** 1 Early Detection, Prevention & Infections Branch, International Agency for Research on Cancer, Lyon, France; 2 Department of Obstetrics and Gynecology, University Teaching Hospital, Lusaka, Zambia; 3 Department of Pathology, University Teaching Hospital, Lusaka, Zambia; 4 Karkinos Healthcare, Kerala Operations, Ernakulam, India

**Keywords:** Medical research, Clinical trial design

## Abstract

Implementing standard-of-care cryotherapy or electrosurgical excision to treat cervical precancers is challenging in resource-limited settings. An affordable technological alternative that is as effective as standard-of-care techniques would greatly improve access to treatment. This randomized controlled trial aims to demonstrate the noninferiority efficacy of a portable, battery-driven thermal ablation (TA) device compared to cryotherapy and electrosurgical excision (large loop excision of transformation zone (LLETZ)) to treat cervical precancer in a screen-and-treat program in Zambia. A total of 3,124 women positive on visual inspection with acetic acid and eligible for ablative therapy were randomized to one of the treatment arms. Human papillomavirus (HPV) testing was performed at baseline and at the follow-up. The primary outcome was treatment success, defined as either type-specific HPV clearance at the follow-up in participants positive for HPV at baseline, or a negative visual inspection with acetic acid test for those who had a negative HPV test at baseline. After a median follow-up of 12 months, treatment success rates were 74.0%, 71.1% and 71.4% for the TA, cryotherapy and LLETZ arms, respectively, thus demonstrating noninferiority (*P* = 0.83). TA was a safe and well-accepted procedure. Only 3.6% of those randomized to TA reported moderate-to-severe pain, compared to 6.5% and 1.9% for the cryotherapy and LLETZ arms, respectively. Thus, our randomized controlled trial demonstrates the safety and efficacy of TA, which is not inferior to cryotherapy or surgical excision.

ClinicalTrials.gov registration: NCT02956239.

## Abstract (web summary)

A randomized controlled trial performed in a screen-and-treat program in Zambia found that a portable, battery-operated thermal ablation device was not inferior to cryotherapy and electrosurgical excision in cervical precancer treatment.

## Main

The ‘screen-and-treat’ approach, in which women who are screen-positive are treated directly without histopathological verification of disease, is a well-accepted protocol in cervical screening programs in low- and middle-income countries (LMICs). The strategy has been endorsed in the latest guideline published by the World Health Organization (WHO) in 2021 because of its simplicity, high acceptance among women and healthcare professionals and the ability to ensure high treatment compliance^1^. Within screen-and-treat settings, thermal ablation (TA) has been used to treat women who are screen-positive on visual inspection with acetic acid (VIA) or high-risk human papillomavirus (HPV) testing following a WHO recommendation^2^. Before this recommendation, cryotherapy and large loop excision of transformation zone (LLETZ) were the accepted standard of care for treating cervical precancers with proven efficacy and safety, the former being more frequently used in LMICs^3^.

While LLETZ is the standard of care in high-resourced settings where treatment is based on colposcopic or histopathological detection of cervical precancer, either ablative treatment methods, that is, TA or cryotherapy, are recommended in a screen-and-treat setting provided certain eligibility criteria are met. These criteria include: the transformation zone (TZ) of the cervix (the area on the cervix that lies between the original and the new squamocolumnar junction) is type I (that is, the squamocolumnar junction is fully visible and is on the ectocervix); the TZ and any lesion, if present, are completely ectocervical; the lesion, if present, covers less than 75% of the ectocervix; and there is no suspicion of invasive cancer^4,5^. Only women ineligible for ablation are advised to undergo LLETZ in such settings. TA has several advantages over cryotherapy: treatment time is much shorter (30–60 s versus 11 min), with less patient discomfort, and the available battery-operated versions are more affordable than cryotherapy, which requires CO_2_ or N_2_O refrigerant gas, whereas TA does not. Difficulties in procuring and maintaining regular supplies of refrigerant gas of appropriate quality are a major deterrent for scaling up cryotherapy in resource-constrained settings^6^. Furthermore, TA equipment is easy to maintain. Multiple overlapping applications to cover a large TZ are feasible with TA but not with cryotherapy. A white paper published by the Clinton Health Access Initiative and Unitaid in 2022 reported an overwhelmingly positive experience in deploying TA in several LMICs across Africa, Latin America and Asia^7^. Despite these advantages, the 2019 WHO recommendation was ‘conditional’ and based on ‘very low certainty in evidence of effects’ given the lack of evidence from any randomized controlled trial (RCT) directly comparing the two treatment strategies in screen-and-treat settings^3^.

To address this evidence gap, the International Agency for Research on Cancer (IARC) collaborated with the University Teaching Hospital Zambia and the University of North Carolina at Chapel Hill (UNC) to improve the prototype of a battery-driven, portable and less expensive thermal ablator, and field-test the new device developed by Liger Medical, who were among the collaborators. We decided to implement the study in Zambia, a country with the highest burden of cervical cancer and human immunodeficiency virus (HIV) infections among women. Moreover, it was feasible to nest the study within the cervical screening program based on VIA that has been ongoing in the country since 2006 (ref. ^8^).

**Extended Data Fig. 1:**
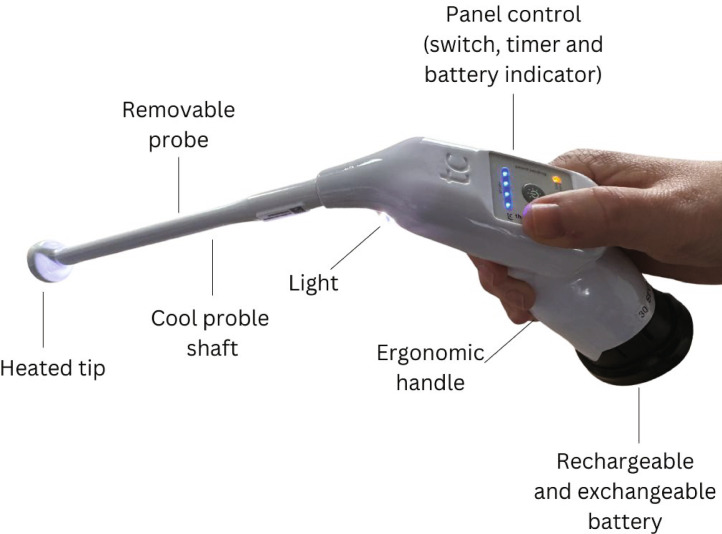
A portable battery-driven thermal ablation device showing different components.

Scaling up TA technology in LMIC settings requires a lighter portable model of thermal ablator (known previously as cold coagulator) but has an obvious advantage in primary care clinics that are space-constrained. Working closely with the manufacturer (Liger Medical), we developed a new version of the thermal ablator customized to the requirements of LMICs. Considering the irregular supply of electricity in LMIC clinics, a rechargeable battery was incorporated in our thermal ablator. The machine was menu-driven and intuitive. A group of colposcopists involved in our study provided feedback to the engineers at Liger Medical on how to improve the ergonomics and functionality of the prototype. Based on such feedback, the device was improved with the addition of a new control panel, a timer with status indicator lights and provision for setting the machine to different treatment times (between 20 and 60 s, with a default of 30 s) (Extended Data Fig. 1). A small removable 12-V battery was incorporated into the handle of the device. The battery can be recharged over 2–3 h; a fully charged battery is enough to complete at least 20 treatment procedures.

We initiated a prospective, randomized, outcome assessor-blind, noninferiority trial in August 2017 to evaluate the efficacy, safety and patient-reported outcomes of the battery-driven thermal ablator compared to cryotherapy and LLETZ, the latter being the gold standard for treating high-grade cervical intraepithelial neoplasia (CIN) (CIN 2 or CIN 3).

## Results

The trial had both a pilot and an extended phase as per the requirements of the funding agency. The pilot phase aimed to generate early efficacy and safety data for the new device and included 750 eligible participants; in the extended phase, more participants were recruited^9^. To achieve an adequate sample size, we had to recruit an additional 791 women in each arm in the extended phase. The only difference between the pilot and extended phase interventions was that the treatment time for TA was reduced from 45 s per application to 30 s. Data from participants in both pilot and extended phases were analyzed together and are reported in the present manuscript.

### Patient disposition

In total 1,042, 1,041 and 1,041 women who were VIA screen-positive were randomly allocated to the TA, cryotherapy and LLETZ arms, respectively. The first participant was recruited on 2 August 2017; the last participant was recruited on 29 September 2022.

**Fig. 1:**
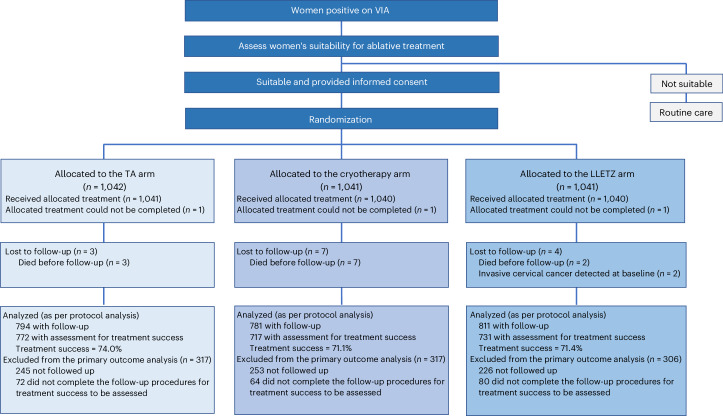
CONSORT diagram. Trial flowchart.

The CONSORT diagram is shown in Fig. 1. Of the 3,124 women recruited, 38.5% (*n* = 1,203) were aged 25 to 29, 39.8% (*n* = 1,244) were aged 30 to 39, 18.8% (*n* = 587) were aged 40 to 49 and 2.9% (*n* = 90) were aged 50 to 59 years (Table 1). The age distribution of participants belonging to the cryotherapy and TA arms was evenly matched while the LLETZ arm had comparably a lower proportion of participants in the 25–29 year age group. Details of other demographic and reproductive factors are provided in Table 1.

**Table 1:** Baseline sociodemographic characteristics, reproductive health history and clinical details of women who were VIA positive randomized to the different treatment arms of the trial

	Randomization arm			
	TA	Cryotherapy	LLETZ	Total
	*n* (%)	*n* (%)	*n* (%)	*n* (%)
Participants recruited, *n*	1,042	1,041	1,041	3,124
Age (years)				
25–29	426 (40.9)	439 (42.2)	338 (32.5)	1,203 (38.5)
30–39	411 (39.4)	389 (37.4)	444 (42.7)	1,244 (39.8)
40–49	181 (17.4)	186 (17.9)	220 (21.1)	587 (18.8)
50–59	24 (2.3)	27 (2.6)	39 (3.7)	90 (2.9)
Participant education				
No formal education	46 (4.4)	40 (3.9)	53 (5.1)	139 (4.5)
Primary	414 (39.8)	429 (41.3)	422 (40.7)	1,265 (40.6)
Secondary	425 (40.9)	393 (37.9)	410 (39.5)	1,228 (39.4)
College or university	155 (14.9)	176 (17.0)	153 (14.7)	484 (15.5)
Occupation				
Homemaker	321 (31.4)	307 (30.1)	265 (26.0)	893 (29.2)
Manual laborer	115 (11.2)	127 (12.4)	128 (12.6)	370 (12.1)
Professional	163 (15.9)	165 (16.2)	152 (14.9)	480 (15.7)
Business	343 (33.5)	348 (34.1)	378 (37.1)	1,069 (34.9)
Other	81 (7.9)	74 (7.2)	96 (9.4)	251 (8.2)
Marital status				
Unmarried	170 (16.3)	176 (16.9)	152 (14.6)	498 (16.0)
Married or cohabiting	693 (66.5)	670 (64.4)	654 (63.0)	2,017 (64.6)
Widowed	63 (6.0)	65 (6.3)	91 (8.8)	219 (7.0)
Separated	116 (11.1)	129 (12.4)	141 (13.6)	386 (12.4)
Residence area				
Urban	878 (84.3)	867 (83.4)	880 (84.7)	2,625 (84.1)
Semi-urban	152 (14.6)	153 (14.7)	150 (14.4)	455 (14.6)
Rural	11 (1.1)	20 (1.9)	9 (0.9)	40 (1.3)
Total number of pregnancies				
None	78 (7.5)	88 (8.5)	72 (6.9)	238 (7.6)
1–2	407 (39.1)	398 (38.2)	386 (37.1)	1,191 (38.1)
3–4	348 (33.4)	367 (35.3)	380 (36.5)	1,095 (35.1)
5+	209 (20.1)	188 (18.1)	203 (19.5)	600 (19.2)
Total number of live births				
None	109 (10.5)	119 (11.4)	106 (10.2)	334 (10.7)
1–2	466 (44.7)	459 (44.1)	465 (44.7)	1,390 (44.5)
3–4	320 (30.7)	333 (32.0)	333 (32.0)	986 (31.6)
5+	147 (14.1)	130 (12.5)	137 (13.2)	414 (13.3)
Last menstruation				
<12 months	985 (95.6)	977 (95.9)	982 (96.4)	2,944 (96.0)
>12 months	45 (4.4)	42 (4.1)	37 (3.6)	124 (4.0)
Baseline HIV status				
Negative	459 (44.8)	456 (44.7)	355 (34.6)	1,270 (41.4)
Positive	566 (55.2)	565 (55.3)	670 (65.4)	1,801 (58.6)
If HIV positive, duration of infection in years				
<2	230 (41.2)	239 (42.6)	250 (38.0)	719 (40.5)
2+	328 (58.8)	322 (57.4)	408 (62.0)	1,058 (59.5)
If HIV positive				
Patient not on ART	9 (1.6)	10 (1.8)	6 (0.9)	25 (1.4)
Patient on ART	555 (98.4)	552 (98.2)	658 (99.1)	1765 (98.6)
HPV test results				
Negative	499 (49.7)	473 (47.0)	423 (42.1)	1395 (46.3)
Positive	506 (50.3)	533 (53.0)	582 (57.9)	1621 (53.7)
HPV type				
HPV16	169 (33.4)	178 (33.4)	215 (36.9)	562 (34.7)
HPV18 or 45	100 (19.8)	110 (20.6)	101 (17.4)	311 (19.2)
HPV31, 33, 35, 39, 51, 52, 56, 58, 59, 66 or 68	237 (46.8)	245 (46.0)	266 (45.7)	748 (46.1)
Baseline HPV and HIV status				
HPV negative, HIV negative	281 (28.4)	288 (29.2)	217 (21.9)	786 (26.5)
HPV negative, HIV positive	161 (16.3)	152 (15.4)	122 (12.3)	435 (14.7)
HPV positive, HIV negative	208 (21.0)	177 (18.0)	201 (20.3)	586 (19.8)
HPV positive, HIV positive	340 (34.3)	369 (37.4)	451 (45.5)	1160 (39.1)

HIV status was known for 3,071 (98.3%) participants, 58.6% (*n* = 1,801) being HIV positive. The majority (59.5%) of women who were HIV positive had an HIV infection for more than 2 years. Almost all women who were HIV positive were on antiretroviral therapy (ART) at the time of recruitment; 55.2% of women allocated to the TA arm were HIV positive, 55.3% of women allocated to the cryotherapy were HIV positive and 65.4% of women allocated to the LLETZ arm were HIV positive (Table 1).

Overall, 53.7% (*n* = 1,621) of the 3,016 participants with a valid HPV report were positive, including 50.3% (*n* = 506), 53.0% (*n* = 533) and 57.9% (*n* = 582) in the TA, cryotherapy and LLETZ arms, respectively (Table 1). At least one-third of women who were HPV positive in each arm (TA, 33.4%; cryotherapy, 33.4%; LLETZ, 36.9%) were HPV type 16 (HIV16) positive. In total, 39.1% (*n* = 1,160) of participants were positive for both HIV and HPV, including 34.3% (*n* = 340), 37.4% (*n* = 369) and 45.5% (*n* = 451) in the TA, cryotherapy and LLETZ arms, respectively.

After excluding 12 deaths (not due to treatment complications or cervical cancer) and two invasive cancers detected at baseline in the LLETZ arm, 3,110 women were eligible for follow-up; 2,386 (76.7%) of them returned for follow-up at 12 months (Table 2). Compliance with follow-up was 76.4% in the TA arm, 75.5% in the cryotherapy arm and 78.2% in the LLETZ arm. The median interval between treatment and follow-up was 12.0 months (interquartile range (IQR) = 10.3–13.5) for women treated with TA, 12.1 months (IQR = 9.0–13.7) for women treated with cryotherapy and 12.0 months (IQR = 10.0–13.3) for women treated with LLETZ.

**Table 2:** Treatment success rates at the 12-month follow-up after treatment based on HPV testing and the VIA results

	Randomization arm				
	TA	Cryotherapy	LLETZ	Total	*P*
	*n* (%)	*n* (%)	*n* (%)	*n* (%)	
Duration of the follow-up after treatment in months					
Median	12.0	12.1	12.0	12.0	
IQR	10.3–13.5	9.0–13.7	10.0–13.3	9.6–13.5	
Follow-up and treatment success					
Randomized participants, *n*	1,042	1,041	1,041	3,124	
Participants eligible for follow-up	1,039 (99.7)	1,034 (99.3)	1,037 (99.6)	3,110 (99.6)	
Participants followed up	794 (76.4)	781 (75.5)	811 (78.2)	2,386 (76.7)	
Participants assessed for treatment success (primary outcome)	722 (69.5)	717 (69.3)	731 (70.5)	2,170 (69.8)	0.825
Participants with no evidence of disease,^a^ *n*	534	510	522	1,566	0.417
Proportion, % (95% CI)	74.0 (70.6–77.1)	71.1 (67.7–74.4)	71.4 (68.0–74.7)	72.2 (70.2–74.0)	
Risk ratio for TA versus cryotherapy (95% CI)^b^	1.03 (0.97–1.08)	1.00			
Risk ratio for TA versus LLETZ (95% CI)^b^	1.00 (0.95–1.05)		1.00		
Treatment success among HIV-negative participants					
Participants assessed for treatment success (primary outcome)	329	317	267	913	
Participants with no evidence of disease^a^	286 (86.9)	266 (83.9)	231 (86.5)	783 (85.8)	0.501
Treatment success among HIV-positive participants					
Participants assessed for treatment success (primary outcome)	383	386	453	1,222	
Participants with no evidence of disease^a^	240 (62.7)	235 (60.9)	284 (62.7)	759 (62.1)	0.834
Treatment success among HPV-positive participants at baseline					
Participants assessed for treatment success (primary outcome)	304	342	361	1,007	
Participants with no evidence of disease^a^	153 (50.3)	172 (50.3)	176 (48.8)	501 (49.8)	0.894
HPV genotype clearance and persistence^b^					
HPV16					
Cleared	62 (60.2)	57 (52.8)	71 (55.5)	190 (56.0)	0.547
Persistent	41 (39.8)	51 (47.2)	57 (44.5)	149 (44.0)	
Total	103 (100.0)	108 (100.0)	128 (100.0)	339 (100.0)	
HPV18 or HPV45					
Cleared	48 (56.5)	54 (60.7)	52 (59.1)	154 (58.8)	0.851
Persistent	37 (43.5)	35 (39.3)	36 (40.9)	108 (41.2)	
Total	85 (100.0)	89 (100.0)	88 (100.0)	262 (100.0)	
HPV31, 33, 35, 39, 51, 52, 56, 58, 59, 66 or 68					
Cleared	114 (51.8)	130 (52.4)	134 (50.2)	378 (51.4)	0.871
Persistent	106 (48.2)	118 (47.6)	133 (49.8)	357 (48.6)	
Total	220 (100.0)	248 (100.0)	267 (100.0)	735 (100.0)	

All data are presented as *n* (%) unless otherwise indicated.

^a^No evidence of disease was defined as either (1) HPV type-specific clearance at 12 months among women positive for the same HPV type at baseline or (2) negative VIA examination at follow-up, if the baseline HPV test was negative.

^b^Adjusted for age at recruitment and baseline HIV status.

Only one study participant had cervical cancer at the follow-up. She belonged to the TA arm, was HIV positive and HPV negative at baseline, and was referred for cancer management. It was not possible to rule out the presence of cancer at the time of recruitment. All women with treatment failure were offered LLETZ. Of the 605 women with type-specific HPV persistence, LLETZ histopathology reports were available for 177 (29.3%) (data not shown in the table), of which 50.3% (*n* = 89) had CIN 2 or CIN 3.

### Noninferiority of treatment response at 12 months

Success of treatment at follow-up for each intervention was considered the primary outcome. This was defined as either type-specific HPV clearance at follow-up in participants positive for high-risk HPV at baseline, or a negative VIA examination (disappearance of the lesion) for those with a negative HPV test and a visible lesion at baseline.

Assessment for the primary outcome was feasible in 69.8% (2,170 of 3,110) of those eligible for follow-up (69.5%, 69.3% and 70.5% in the TA, cryotherapy and LLETZ arms, respectively) (Table 2).

The proportion of participants assessed for the primary outcome with treatment success as per our prespecified criteria was 74.0% (534 of 722) in the TA arm, 71.1% (510 of 717) in the cryotherapy arm and 71.4% (522 of 731) in the LLETZ arm. The treatment success rate in the TA arm was not inferior to those in the cryotherapy and LLETZ arms. The lower bounds of the 95% confidence intervals (CIs) of risk ratios for both TA and cryotherapy (0.97–1.08) and TA versus LLETZ (0.95–1.05) were above 0.61, that is, the noninferiority criterion derived from the sample size assumptions.

### Treatment side effects and complications

Secondary outcomes were treatment side effects and complications, including patient-reported pain and discomfort, as well as the overall satisfaction level (Table 3). The most frequent side effect, as reported by the nurse providing treatment, was lower abdominal and pelvic pain or cramps (or both), which was experienced by 59.6% (*n* = 621) of women undergoing TA, 61.6% (*n* = 641) of women undergoing cryotherapy and 50.0% (*n* = 520) of women undergoing LLETZ. The difference was statistically significant (*P* = <0.001). The nurses also documented that moderate-to-severe pain was reported by 3.6% of women treated with TA, 6.5% of women treated with cryotherapy and 1.9% of women treated with LLETZ. Bleeding was observed in 0.5%, 0.3% and 0.8% of patients undergoing TA, cryotherapy and LLETZ, respectively. The assigned treatment was completed in all except one patient in each arm.

**Table 3:** Side effects reported by the treating nurse during or immediately after treatment

	Randomization arm				
	TA	Cryotherapy	LLETZ	Total	*P*
	*n* (%)	*n* (%)	*n* (%)	*n* (%)	
Participants treated, *n*	1,042	1,041	1,041	3,124	
Side effects					
Overall	624 (59.9)	644 (61.9)	524 (50.3)	1,792 (57.4)	<0.001
Pain or cramps	621 (59.6)	641 (61.6)	520 (50.0)	1,782 (57.0)	<0.001
Mild	584 (56.0)	573 (55.0)	500 (48.0)	1,657 (53.0)	
Moderate	37 (3.6)	64 (6.1)	20 (1.9)	121 (3.9)	
Severe	0 (0)	4 (0.4)	0 (0)	4 (0.1)	
Bleeding	5 (0.5)	3 (0.3)	8 (0.8)	16 (0.5)	
Vaginal burning	2 (0.2)	0 (0)	3 (0.3)	5 (0.2)	
Vasovagal reaction	0 (0)	1 (0.1)	0 (0)	1 (0)	
Anaphylactic reaction	0 (0)	1 (0.1)	1 (0.1)	2 (0.1)	
Others	1 (0.1)	7 (0.7)	1 (0.1)	9 (0.3)	
Procedure could be completed	1,041 (99.9)	1,040 (99.9)	1,040 (99.9)	3,121 (99.9)	

Thirteen serious adverse events (including 12 deaths) were reported. These included three participants in the TA arm, eight participants in cryotherapy arm and two participants in the LLETZ arm. None of these events were associated with any of the treatment procedures.

### Patient-reported outcomes

Patient-reported pain intensity or discomfort and treatment satisfaction level at treatment completion was recorded immediately after treatment and again 2 weeks later (Table 4). Most patients (98.6%) reported either no pain or minimal pain (score 1–3 on a 9-point rating scale) after treatment completion. The proportion of patients reporting any pain was slightly higher in the cryotherapy arm (37.4%) compared to TA (35.0%) or LLETZ (30.8%) arm. Almost all patients (99.2%), irrespective of treatment type, were highly satisfied with the service provided.

**Table 4:** Patient-reported outcomes: pain intensity and satisfaction level reported at the end of the treatment visit and 2 weeks later

	Randomization arm				
	TA	Cryotherapy	LLETZ	Total	*P*
	*n* (%)	*n* (%)	*n* (%)	*n* (%)	
At the end of the treatment visit					
Participants assessed, *n*	1,042	1,041	1,041	3,124	
Level of pain or discomfort felt (score ranging from 1 to 9)^a^					
1 (no pain)	673 (64.6)	649 (62.3)	719 (69.1)	2,041 (65.3)	0.035
2–3 (least pain)	351 (33.7)	376 (36.1)	314 (30.2)	1,041 (33.3)	
4–6	10 (1.0)	10 (1.0)	6 (0.6)	26 (0.8)	
7–9 (worst pain)	3 (0.3)	3 (0.3)	0 (0)	6 (0.2)	
Level of satisfaction with the service provided (score ranging from 1 to 9)^b^					
1–3 (least satisfied)	4 (0.4)	3 (0.3)	3 (0.3)	10 (0.3)	0.877
4–6	2 (0.2)	3 (0.3)	1 (0.1)	6 (0.2)	
7–9 (highly satisfied)	1,033 (99.1)	1,031 (99.0)	1,035 (99.4)	3,099 (99.2)	
Would recommend the screening procedure to others					
Yes	1,038 (99.6)	1,036 (99.5)	1,038 (99.7)	3,112 (99.6)	0.440
No	3 (0.3)	2 (0.2)	3 (0.3)	8 (0.3)	
Cannot say	1 (0.1)	3 (0.3)	0 (0)	4 (0.1)	
Two weeks after treatment					
Participants assessed	933	933	941	2,807	
Level of pain or discomfort felt (score ranging from 1 to 9)					
1 (no pain)	899 (96.4)	882 (94.5)	888 (94.4)	2,669 (95.1)	0.383
2–3 (least pain)	26 (2.8)	43 (4.6)	37 (3.9)	106 (3.8)	
4–6	0 (0)	1 (0.1)	2 (0.2)	3 (0.1)	
7–9 (worst pain)	1 (0.1)	1 (0.1)	1 (0.1)	3 (0.1)	
Level of satisfaction with the service provided (score ranging from 1 to 9)					
1–3 (least satisfied)	1 (0.1)	0 (0)	1 (0.1)	2 (0.1)	0.737
4–6	0 (0)	1 (0.1)	1 (0.1)	2 (0.1)	
7–9 (highly satisfied)	924 (99.0)	925 (99.1)	929 (98.7)	2,778 (99.0)	
Would recommend the screening procedure to others					
Yes	926 (99.2)	926 (99.2)	928 (98.6)	2,780 (99.0)	0.530
No	1 (0.1)	2 (0.2)	2 (0.2)	5 (0.2)	
Cannot say	6 (0.6)	5 (0.5)	11 (1.2)	22 (0.8)	

^a^Information missing for ten participants.

^b^Information missing for nine participants.

Assessment of discomfort or pain and the satisfaction level of patients was carried out using telephonic surveys 2 weeks after treatment; it was completed in 933 women each in the cryotherapy and TA arms and 941 women in the LLETZ arm (Table 4). Only one patient treated with TA, two patients treated with cryotherapy and three patients treated with LLETZ reported moderate-to-severe pain (score 4–9), while 96.4%, 94.5% and 94.4% of patients in the TA, cryotherapy and LLETZ arms, respectively, reported no residual pain. There was no difference in patient-reported pain across the three treatment arms 2 weeks after treatment. The level of satisfaction with all types of treatment was persistently high throughout the study. Overall, 99.2% of the study participants were highly satisfied (scored 7–9 on a 9-point rating scale) with the service at the end of the treatment visit, without any significant difference (*P* = 0.877) between the three study arms. The proportion of participants with a satisfaction score between 7 and 9 was 99% overall 2 weeks after treatment.

### Baseline histopathology of women treated with LLETZ

None of the study participants had punch biopsies taken before treatment. However, histopathology reports were available for 1,000 women (of 1,041) treated using LLETZ. Histopathological reports were normal in 421 (42.1%). CIN 1, CIN 2 and CIN 3 lesions were detected in 184 (18.4%), 131 (13.1%) and 262 (26.2%) women, respectively (data not shown in the table). The prevalence of CIN 2+ lesions was 31.8%, 39.5% and 48.8% for the 25–29, 30–39 and 40–59 age groups, respectively. Invasive cancer that had not been suspected at the initial VIA was detected in two (0.2%) patients. Both were HIV positive. Nearly half (49.5%) of the women who were HIV positive treated with LLETZ had CIN 2 or worse disease as determined by histopathological analysis.

### Subgroup analyses

We performed post hoc analyses of treatment success categorized according to baseline HPV and HIV status in the pilot and extended phases, using VIA as the only treatment outcome.

#### Treatment success according to HPV status, HPV type and HIV status at baseline

We analyzed treatment response stratified according to baseline HIV status, HPV status and HPV genotype (Table 2). In women who were HIV negative, the overall treatment success rate was 85.8% (783 of 913), without any significant difference observed between the study arms (cryotherapy: 83.9%; TA: 86.9%; LLETZ: 86.5%; *P* = 0.5). The treatment success rate was substantially lower in women who were HIV positive (*n* = 1223), irrespective of treatment type (overall: 62.1%; cryotherapy: 60.9%; TA: 62.7%; LLETZ: 62.7%). No statistically significant difference was observed between the treatment methods (*P* = 0.83) among the HIV-positive women.

Type-specific HPV clearance was observed in 49.8% (501 of 1,007) of women who were HPV positive at baseline, with no significant difference observed between the study arms (cryotherapy: 50.3%; TA: 50.3%; LLETZ: 48.6%; *P* = 0.89). Treatment success (type-specific clearance) was observed in 56.0% (190 of 339) of women who were HPV16 positive, 58.8% (154 of 263) of women who were HPV18- and HPV45 positive and 51.4% (378 of 735) of women positive for other high-risk types; no statistically significant difference was observed across the study arms (Table 2).

#### Treatment success in the pilot and extended phases

For the primary outcome, sensitivity analyses performed separately on data from the pilot and extended phases resulted in similar treatment success. Treatment success in the TA arm was not inferior to that of the cryotherapy (risk ratio = 1.06; 95% CI = 0.93–1.21) or LLETZ (risk ratio = 0.99; 95% CI = 0.88–1.11) arm in the pilot phase (Table 5). Noninferiority of treatment success with TA compared to cryotherapy (risk ratio = 1.01; 95% CI = 0.96–1.07) and LLETZ (risk ratio = 1.02; 95% CI = 0.95–1.08) was demonstrated in the analysis of the extended phase data.

**Table 5:** Treatment success rates after treatment at follow-up based on HPV testing and VIA results separated according to the initial 750 participants and the participants in the extended phase

	Randomization arm			
	TA	Cryotherapy	LLETZ	Total
	*n* (%)	*n* (%)	*n* (%)	*n* (%)
Initial 750 participants recruited for the pilot phase				
Duration of follow-up after treatment in months				
Median	6.1	6.0	6.0	6.0
IQR	6.0–6.6	6.0–6.7	6.0–6.8	6.0–6.6
Follow-up and treatment success				
Randomized participants, *n*	250	250	250	750
Participants eligible for follow-up	249 (99.6)	247 (98.8)	249 (99.6)	745 (99.3)
Participants followed up	213 (85.5)	213 (86.2)	217 (87.1)	643 (86.3)
Participants assessed for treatment success (primary outcome)	211 (84.7)	212 (85.8)	215 (86.3)	638 (85.6)
Participants with no disease,^a^ *n*	133	120	141	394
Proportion, % (95% CI)	63.0 (56.1–69.6)	56.6 (49.6–63.4)	65.6 (58.8–71.9)	61.8 (57.9–65.5)
Risk ratio for TA versus cryotherapy (95% CI)^b^	1.06 (0.93–1.21)	1.00		
Risk ratio for TA versus LLETZ (95% CI)^b^	0.99 (0.88–1.11)		1.00	
Among participants recruited in the extended phase				
Duration of follow-up after treatment in months				
Median	12.3	12.6	12.3	12.3
IQR	12.0–14.4	12.0–14.7	12.0–14.0	12.0–14.4
Follow-up and treatment success				
Randomized participants, *n*	792	791	791	2,374
Participants eligible for follow-up	790 (99.7)	787 (99.5)	788 (99.6)	2,365 (99.6)
Participants followed up	581 (73.5)	568 (72.2)	594 (75.4)	1,743 (73.7)
Participants assessed for treatment success (primary outcome)	511 (64.7)	505 (64.2)	516 (65.5)	1,532 (64.8)
Participants with no disease,^a^ *n*	401	390	381	1,172
Proportion, % (95% CI)	69.0 (74.7–82.0)	68.7 (73.3–80.8)	64.1 (69.8–77.6)	67.2 (74.3–78.6)
Risk ratio for TA versus cryotherapy (95% CI)^b^	1.01 (0.96–1.07)	1.00		
Risk ratio for TA versus LLETZ (95% CI)^b^	1.02 (0.95–1.08)		1.00	

All data are presented as *n* (%) unless otherwise indicated.

^a^No evidence of disease was defined as either (1) HPV type-specific clearance at 12 months among women positive for the same HPV type at baseline or (2) negative VIA test at the follow-up, if the baseline HPV test was negative.

^b^Adjusted for age at recruitment and baseline HIV status.

#### Treatment success assessed according to the VIA results alone

To determine the success rate of treatment in a routine clinical setting in Zambia, we assessed the outcome on the basis of the initial VIA findings alone (Extended Data Table 1). Based on the initial VIA positivity, the posttreatment VIA negativity rate was higher in women without HIV (89.0% versus 81.9%). Although women who were HIV positive had lower success rates than women who were HIV negative, there was no notable difference between the treatment arms overall and in women who were HIV positive.

## Discussion

Our RCT nested into an ongoing cervical screening program in Zambia demonstrated that the treatment success rate of TA performed by trained nurses was not inferior to those observed for cryotherapy and LLETZ. The safety profiles of all three treatment methods were comparable. The outcomes of our RCT implemented within a real-world setting strengthens the existing evidence base for national and international organizations to recommend TA to treat cervical precancers, especially in ‘screen-and-treat’ or screen, triage and treat scenarios with higher degrees of certainty.

According to a prespecified analysis, we published the results of the pilot component of this study in 2019, having recruited 250 women were were VIA positive for each treatment arm^8^. Using the same criteria to measure treatment success, as described in this article but with a shorter follow-up (6 months), we observed no significant difference (*P* = 0.31)^9^ between the three treatment approaches; 64% of women treated with TA, 60% of women treated with cryotherapy and 67% of women treated with LLETZ had a successful treatment. Almost all women in the pilot study across all three treatment arms reported no or minimum levels of discomfort with their treatment, either immediately after or within 2 weeks of treatment. The treatment time of TA was reduced from 45 to 30 s per application in the extended phase of this trial. Even then, the treatment success of TA was not inferior to those of the other treatment techniques, when compared to the pilot study.

In this study, we included patient-reported outcomes to assess the impact of treatment from the patient’s perspective, which is increasingly being recognized by regulators, healthcare professionals and policy-making bodies as a valuable tool to collect patient-centered data^10^. Using both patient-reported and healthcare provider-reported outcomes, we showed that TA has an excellent safety profile. The intensity of pain or discomfort experienced was a little higher with cryotherapy and the lowest among women treated with LLETZ because they received local anesthesia. Histopathology undertaken after treatment for the LLETZ arm showed that there was a risk, albeit very small, of inadvertently ablating an invasive cancer, especially if the woman was HIV positive.

In their meta-analysis of 13 studies evaluating TA, Dolman et al.^11^ reported a high cure rate of 95% (95% CI = 92% to 98%) when treating CIN 2 and CIN 3 lesions, as well as a good safety profile. Only one of the studies included in their meta-analysis was from an LMIC in Asia. None had any data on women who were HIV positive. The results from our large prospective RCT provide much needed evidence on the noninferiority of efficacy of TA compared to cryotherapy or LLETZ when treating type 1 lesions, that is, ectocervical TZs from a real-world screen-and-treat setting with high HIV prevalence among women undergoing cervical screening.

Furthermore, our study highlights the high treatment failure rates observed in women who are HIV positive, irrespective of the treatment used, which is a matter of great concern. Like our study, a previous RCT conducted in Kenya that compared cryotherapy to LLETZ in 354 women who were HIV positive, observed type-specific HPV persistence in 61% and 49% of participants treated with cryotherapy and LLETZ, respectively^12^. The reasons for these high failure rates and the clinical significance of type-specific HPV infection persistence in women who are HIV positive need to be investigated further. We could not include CD4 counts or viral load in the treatment of women who are HIV positive included in our trial, which may have also influenced treatment outcomes. A new approach to the prevention of cervical cancer in women who are HIV positive is urgently needed. Perhaps, prophylactic TA of the TZ at the initial discovery of HIV should be considered, when the TZ is probably smaller, ectocervical and when treatment is likely to be uncomplicated. Several studies reported that HPV-induced high-grade cervical precancers and cancers originate from a small discrete cell population (junctional cells) localized to the squamocolumnar junction of the cervix^13^. These have a unique gene expression signature and are not regenerated after treatment. Thus, ablation of these totipotent stem cells in young women newly diagnosed with HIV may significantly reduce the future risk of cervical neoplasia^14^.

The strengths of the study were that the same assessment technique was used in all three study arms, assessors were blinded to randomization and no significant difference between treatment methods was observed, even when we analyzed the data of participants whose assessment was based only on HPV testing. However, our RCT has limitations. First, given the challenges of performing quality-assured histopathology in Zambia, we decided to adopt a pragmatic approach in recruiting study participants and assessing treatment success that closely resembles the real-world screen-and-treat paradigm. Disease status at recruitment was established based on VIA examination only, without histopathology confirmation. To estimate the prevalence of CIN 2 and CIN 3 in each study arm, we performed histopathological evaluation of the specimens excised in the arm randomized to LLETZ. This showed that at least one-third of participants had CIN 2 or CIN 3 lesions. Even then, the results are applicable to a screen-and-treat setting only and may not be extrapolated to assess the efficacy of these techniques to treat histopathology-established CIN 2 and CIN 3 lesions.

Another limitation is that the assessment of treatment success at 1 year was based on either type-specific clearance of HPV or the disappearance of the lesion at VIA (in women who are HPV negative), rather than colposcopic or histopathological verification, as often used in studies of treatment efficacy. For this reason, the success rates of TA and the other two techniques in our study, even in women who are HIV negative, were lower than the high success rates (>90%) reported in previous meta-analyses of trials that used histopathological verification of CIN 2 and CIN 3 lesions both at baseline and follow-up^11,15–17^. We selected this outcome to measure treatment success because clearance of high-risk HPV 12 months after treatment is a reliable ‘test of cure’ for CIN 2 and CIN 3 lesions, with many studies reporting a nearly 100% negative predictive value^18–20^. On the other hand, type-specific persistence of HPV at 12 months had a positive predictive value of 43% to detect residual and recurrent CIN 2+ lesions in the Kenyan RCT comparing cryotherapy to LLETZ^12^. We also observed that nearly half of patients with type-specific HPV persistence at follow-up had CIN 2+ lesions. Thus, HPV status was a good predictor of residual and recurrent disease at the 12-month follow-up in a setting where colposcopic and histopathological verification of disease status was practically difficult. Our study was not designed to capture the possible long-term adverse impact of cervical precancer treatment on fertility and early pregnancy outcomes reported by other studies that evaluated cryotherapy and LLETZ^21^. Not reporting such an important outcome is a limitation of our study that needs to be addressed in future studies.

The coronavirus disease 2019 (COVID-19) pandemic did not have any significant impact on the study except that we had to withhold the recruitment of new participants for nearly 6 months during and after the lockdown period. Site staff ensured the follow-up of patients already treated by providing travel support and creating safe COVID-19-free examination clinics.

To conclude, the present study confirmed that a portable TA device was an acceptable, effective and safe method to treat a selected cervical type 1 TZ within a screen-and-treat setting. TA has several important advantages over cryotherapy, such as the need for expensive consumables not being required, the ability to be used in settings without electricity and the reduced treatment time. The battery-driven thermal ablator developed and evaluated through our IARC project is already being widely used in India, China and several sub-Saharan African countries because of its ease of use, low running costs and portability^6,22,23^. The latest guideline for cervical screening in Zambia published in 2023 incorporated treatment with a battery-operated thermal ablator^24^. It has significantly reduced the need for LLETZ, which requires expensive equipment and tools, highly skilled providers, administration of local anesthesia and a hospital setting. Our data strengthen the evidence base for healthcare professionals and program managers to scale up TA technology.

## Methods

### Study design

The prospective, randomized, three-arm, partially blind, noninferiority trial to evaluate three techniques for treating the cervical TZ in a screen-and-treat setting was launched in August 2017 in Lusaka, Zambia. TA using a portable, battery-driven device manufactured by Liger Medical was compared to cryotherapy and LLETZ in this trial; all procedures were performed on an outpatient basis. The study was implemented in two phases: a pilot phase and an extended phase. Before launching the pilot study, the study team extensively interacted with the engineers at Liger Medical to help them improve a prototype version of the TA device. The improved device was used for the trial.

In the pilot phase, 250 eligible women were randomly recruited to each of the three treatment arms and were followed up after 6 months. The safety and efficacy data from the pilot phase were reviewed by a data safety monitoring board (DSMB). As the efficacy of TA using the new device was not inferior to the other two techniques and as TA had a safety profile similar to the other two techniques, the DSMB allowed continuation of the trial to the extended phase with the following suggestions: (1) to reduce the application of the TA probe from 45 to 30 s; (2) to extend the follow-up time from 6 to 12 months; and (3) to carry out a formal sample size estimation based on the efficacy data of the pilot phase.Sample size was recalculated to recruit additional women to each arm in the extended phase of the trial. The women recruited to the pilot study were included to achieve the required sample size. Recruitment to the extended phase was initiated on 2 January 2019.

### Study target population

The study was nested in the ongoing cervical screening program in Zambia. Screening-eligible women attending primary health clinics (Chawama Level 1 Hospital, Chipata Level 1 Hospital, Kanyama Level 1 Hospital, Levy Mwanawasa Hospital, Matero Clinic) and the University Teaching Hospital in Lusaka, Zambia were the target population for our study. Many of these women attend the clinics to receive ART. All women of an eligible age attending the clinics are routinely counseled to participate in the Zambian national cervical screening program. The clinics follow the Zambian national protocol, which recommends that women aged 25–49 should be tested using VIA and that women who are VIA positive should be offered immediate treatment (screen-and-treat approach). Women aged up to 59 years who have never been screened before are also offered VIA. Both VIA screening and treatment are performed by nurses trained as part of the cervical cancer prevention 2-week training program, including didactic and hands-on clinical mentoring. As part of quality control, trained nurses are encouraged to use distance consultation of local doctors or gynecologists using the electronic transfer of digital photographs of the screened cervix to make appropriate treatment or referral decisions^8^.

Women willing to undergo screening are examined by a trained nurse at the clinic to perform VIA. Women who are VIA positive are further assessed for eligibility for ablative treatment using the following criteria: the TZ is type 1 (the entire squamocolumnar junction is visible at the external os or ectocervix); the acetowhite area on the TZ does not occupy more than 75% of the ectocervix; the acetowhite area does not extend to the endocervix or vagina; and there is no suspicion of invasive cervical cancer on visual examination.Women who were VIA positive and also eligible for ablative treatment were approached to participate in our study.

### Selection and recruitment of study participants

Women who were VIA positive who fulfilled the criteria for suitability for ablative treatment and showed interest to participate in the study were further assessed by a trained social worker for the following inclusion and exclusion criteria before she could initiate the informed consent process. The inclusion criteria included: age between 25 and 59 years; positive VIA test; eligible for ablative treatment based on the criteria described above; additionally, the TZ could be covered by a single application of the largest cryotherapy probe. Exclusion criteria included: pregnant at the time of recruitment; and not in a position to provide voluntary informed consent because of mental illness or other medical conditions.

During the consent process, the social worker explained the objectives, treatment procedures, follow-up requirements, benefits and harms of participating in the study and responded to any queries. Each woman willing to participate provided written consent before her recruitment to the study was finalized.

### Randomization and masking

The recruited women were randomly assigned to one of the three treatment groups in 1:1:1 ratio. Before the study was launched, a randomization list was generated by the IARC data manager using the random function in Excel to allocate a treatment to each participant’s ID. This list was not shared with investigators in Zambia. An IARC study coordinator inserted the treatment allocation printed on the participant’s barcode sheets with a specific color code in opaque sealed envelopes with only the randomization sequence written on the envelopes. These envelopes were mailed to the site coordinator in Zambia, who arranged them serially according to the sequence number. The social worker telephoned the site coordinator after confirming participation of an eligible woman to know her treatment allocation. The social worker conveyed the treatment allocation to the treating nurse. Once allocated to a particular treatment arm, the treatment modality could not be changed. Blinding of either the participant or the treating nurse was not feasible.

### Sample collection for HPV testing

The cervical sample from every woman undergoing initial VIA before application of acetic acid was collected using a cytobrush. The samples obtained from women recruited to the study were sent to the laboratory at the University Teaching Hospital, Lusaka for high-risk HPV testing. The test results were made available on a later date and did not change clinical management.

### Treatment procedures

Treatment was provided on the day of randomization at the recruiting clinic by nurses trained to perform all three treatment techniques. Some of the more difficult LLETZ procedures were performed by gynecologists at the clinic. Five percent acetic acid was applied to the cervix to delineate the lesion and the TZ before treatment. The cervix was infiltrated with local anesthetic before LLETZ. TA or cryotherapy was performed without any anesthesia.

A Liger TA device was used to perform TA. The probe was heated to 100 °C and applied to the cervix for 45 s in the pilot phase to treat the initial 250 women recruited to the arm. Treatment time was reduced to 30 s for all women recruited to the TA arm in the extended phase. Overlapping applications were made to cover a large TZ.

Cryotherapy was performed using a cycle of 3-min freezing, 5-min thawing and 3 min of repeat freezing. The probe was applied only once. The refrigerant gas used was nitrous oxide.

For the LLETZ procedure, an appropriately sized loop was selected to remove the entire TZ, if possible. However, multiple passes were used when required. Bleeding after excision was controlled by cauterizing the base of the excised area with a ball diathermy electrode followed by application of Monsel’s paste.

### Posttreatment procedures

Once the procedure was completed, the woman was assessed for any side effects like pain, bleeding and vaginal injury by the treating nurse. No antibiotics were given after treatment.

A social worker explained to the woman that she would be having vaginal discharge for 1–2 weeks, which might be blood-stained at times. She was advised to avoid penetrative vaginal sex for 2 months and report to the facility or telephone the study coordinator if she had any of the following symptoms within 2 weeks of treatment: excessive vaginal bleeding, especially with the passage of clots; foul-smelling excessive vaginal discharge; and pain in the lower abdomen with a temperature exceeding 100 °F.

The social worker also explained the follow-up plan. She informed the woman about a telephone interview to be conducted 2 weeks after treatment to check for any side effects and complications and to receive feedback from the woman regarding how satisfied she was with treatment. The social worker also responded to any queries the woman might have.

At the end of the visit, the social worker conducted the patient-reported outcome survey. The woman was asked to score the level of discomfort and pain she experienced during on immediately after treatment on a visual analog scale ranging from 1 (no pain at all) to 9 (pain was so severe that she wanted the treatment to be stopped). She was also asked to rate her overall satisfaction with the service and experience on a visual analog scale of 1 (highly satisfied) to 9 (least satisfied). The woman was advised to return to the clinic for a checkup after 6 months in the pilot phase and after 12 months in the extended phase.

### Quality assurance of VIA and treatment

The nurses routinely collected and stored cervical images for future quality assurance after each VIA procedure. Quality assurance sessions were held routinely on a monthly basis; 10% of the cases seen by each nurse over the past month were presented by the nurse and reviewed by the entire cervical cancer prevention team of clinicians. During the sessions, each nurse was quizzed on TZ types, presence or absence of acetowhite lesions, selection of treatment (TA versus LLETZ), presence of lesions suspicious for invasive cancer and adequacy of the LLETZ procedure in excising the entire lesion. At the end of each session, the principles of VIA and the treatment of precancerous lesions were reviewed with a focus on areas where weaknesses were noted.

### Monitoring of adverse events

Every participant was advised to attend the clinic or call the dedicated telephone number of the study coordinator if she had any of the symptoms mentioned earlier or if she required medical consultation or hospitalization because of any reason within 2 weeks after treatment. The study coordinator telephoned every participant 2 weeks after treatment to check their well-being and verify if any medical consultation was required in the intervening period. After the first call, the coordinator called the woman every 3 months until her follow-up clinic visit to check if any major illness or hospitalization occurred in the intervening period. The phone number of the partner or husband or a close relative was obtained from each study participant. This number was used if the participant could not be reached on her mobile phone.

All adverse events were recorded in the project database. Serious adverse events were notified to IARC within 24 h of receiving the information.

### Posttreatment follow-up

The social worker conducted a telephone interview with each participant 2 weeks after treatment to repeat the patient-reported outcome survey and check if the woman had any clinic visit or hospitalization in the intervening period. Women were contacted over the telephone approximately 1 month before the scheduled follow-up visit to remind them about the checkup.

At the follow-up visit (which was 6 months after treatment for the pilot phase and 12 months after treatment for the extended phase), a nurse collected a cervical swab for high-risk HPV detection testing and proceeded to perform the VIA. The results of HPV testing were communicated to the women over the telephone and women who were HPV positive were recalled to the clinic. Women with type-specific persistence of infection or positive VIA findings were offered LLETZ. Women not willing to undergo LLETZ were offered ablative treatment. Women negative on the HPV test and VIA were advised to continue with routine screening.

### Detection of high-risk HPV

All baseline cervical samples were tested in the laboratory for high-risk HPV infection; only women with a positive baseline HPV result had their follow-up cervical samples tested. All cervical samples were analyzed using the Xpert HPV test (Cepheid), which detects 14 high-risk HPV types and provides genotype information in three separate channels for HPV16, HPV18 and HPV45 combined, and the remaining 11 HPV types combined (HPV31, 33, 35, 39, 51, 52, 56, 58, 59, 66 and 68). Testing was performed at the University Teaching Hospital, Lusaka.

### Outcomes

The primary outcome of interest was treatment success. As per a prespecified analysis plan, treatment success at the 12-month follow-up was defined as either HPV type-specific clearance in participants positive for high-risk HPV at baseline, or negative VIA testing if the baseline HPV test was negative. Secondary outcomes included treatment side effects and complications, and patient-reported outcomes (pain and discomfort and satisfaction level) immediately after treatment and 2 weeks later. As an exploratory outcome, we analyzed the histopathology results of women undergoing LLETZ to estimate overtreatment in a screen-and-treat setting. We carried out post hoc subgroup analyses stratified according to HIV status and the baseline results of HPV testing for the pilot versus extended phase.

### Sample size estimation

In the pilot phase of the study, 250 women were allocated to one of the treatment arms. Successful completion of the pilot was a requirement specified by the funding agency to support the extended phase of the study. Sample size was calculated before the extended phase using data from the pilot phase. To demonstrate noninferiority in the treatment success rates, we used the following assumptions: the treatment success rate in the cryotherapy (comparator) arm to be 55% (treatment success rate in the cryotherapy arm at the 6 months follow-up at the end of the pilot phase was 55%). We used this as a conservative treatment success rate at 12 months; difference in treatment success rates between TA and cryotherapy to be 5% (at the end of the UH2 pilot phase, a 5% difference in treatment success rates at the 6-month follow-up was observed, with a 60% treatment success rate in the TA arm); a noninferiority margin of 4%; an annual follow-up default rate of 30%; an 80% power; and a 2.5% significance level. To account for the three-arm multiple comparisons and for the planned subanalyses stratified according to HIV status, a 0.00417 (0.025/3/2) significance level was used in the calculations.We estimated that a total sample size of 3,123 women who were VIA screen-positive would be required to confidently answer the primary questions posed in this study. This was equally randomized to the three arms, with 998 women per arm. The pilot study recruited 750 participants. Based on the sample size estimation, we planned to recruit an additional 2,373 participants in the extended phase of the RCT.

The original study protocol did not have a plan to incorporate a pilot study. The estimated sample size for the study was 4,434 women to be distributed equally among the three arms. The funding agency (National Cancer Institute/National Institutes of Health (NIH)) advised us to split the study into a pilot and an extended phase. After completion of the pilot phase, we reestimated the required sample size using data from the pilot phase. The revised total sample size was 3,873 (including the 750 women who participated in the pilot phase). This explains the discrepancy in sample size between the protocol and the present article.

### Statistical analysis

Study data were collected and managed using Research Electronic Data Capture hosted at IARC, and analyzed using STATA v.17.0 (Stata-Corp). The participant baseline characteristics and follow-up status, and primary and secondary outcomes, were shown as proportions; the comparison between the three study arms was determined using the Pearson chi-squared test.

Multiple imputation was used to cater for data missingness in the outcomes and some of the explanatory variables. The effect of the treatment arm on the primary outcome was evaluated using the protocol analysis (in which only participants who followed the study procedures as per the assigned treatment arm were included). Effect estimates were provided as relative risks together with their 95% CIs obtained from the generalized linear models for the binomial regression using the log link function. The multivariate regression model involved adjustment for age and baseline HIV status. The regression analyses were further adjusted for clustering on a combined phase–study center variable to cater for the possible correlation of responses within the study phase and center (that is, the responses may have been independent between but not necessarily within study centers). Statistical significance between the effect estimates of the TA arm compared to each of the other two treatment arms was inferred if the lower bound of the CI was greater than 0.61. This criterion was based on the assumptions for the sample size and based on the difference in treatment success and the margin of noninferiority. Post hoc subgroup analyses were performed in which the assessment of the primary outcome was stratified according to baseline HPV and HIV status, and according to the pilot and extended phases. An additional post hoc subgroup analysis was done using VIA only in the definition of treatment success.

### Ethics and inclusion statement

The collaborators at the University Teaching Hospital, Zambia had major roles in study design, study implementation, data analysis and manuscript preparation. G.P.P., the principal investigator from Zambia, obtained a visiting scientist position at IARC to work with IARC scientists to plan the study, determine the statistical analysis plan and the sample size. Once the study was approved by the funding agency, a meeting was organized in Zambia to plan the details of the study implementation, to ensure the safety, well-being and data privacy of the study participants and to conduct the study according to good clinical practice. We planned to implement the study in multiple primary care clinics in Lusaka, Zambia to ensure that the study outcomes could be quickly adopted and scaled up in the ongoing VIA-based cervical screening program. Building the capacity of different healthcare professionals (nurses, gynecologists, pathologists) and social workers in Zambia was one of the major objectives of this collaborative study. Accordingly, multiple training programs were organized in Zambia. Collaborators from Zambia visited IARC from time to time and especially when the pilot and extended phase data were jointly analyzed. To ensure the safety and well-being of the trial participants, all study staff were trained in good clinical practice. A dedicated mobile phone number was made available 24/7 for participants to contact the study coordinator regarding any problem. The study coordinator maintained regular telephone contact with the participants, especially during the COVID-19 outbreak, to ensure that their follow-up took place in a safe environment. The DSMB consisted of five members, including a lay person, to monitor the study from time to time. The renowned researcher and gynecological oncologist from South Africa, L. Denny, chaired the DSMB. Because the study involved participation of a large number of women who were HIV positive, an expert in managing patients who were HIV positive was a DSMB member.

### Ethics approval

The study was reviewed and approved by the research ethics committees at IARC (IEC 2016-01 approved on 4 March 2016) and UNC (MH/101/23/10/1 approved on 10 July 2017). Each study participant signed a written informed consent. An information leaflet was shared with the participants and the contents were explained by the study coordinator before obtaining consent.

### Reporting summary

Further information on research design is available in the Nature Portfolio Reporting Summary linked to this article.

## Online content

Any methods, additional references, Nature Portfolio reporting summaries, source data, extended data, supplementary information, acknowledgements, peer review information; details of author contributions and competing interests; and statements of data and code availability are available at 10.1038/s41591-024-03080-w.

## Supplementary information

Reporting Summary

## Figures and Tables

**Extended Data Table 1:**
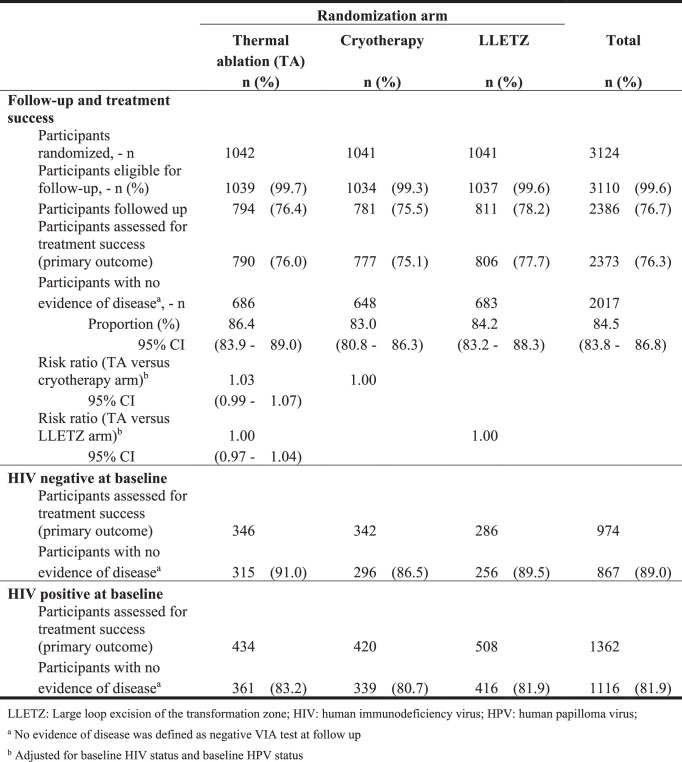
Treatment success rates after treatment at the follow-up based only on the VIA results

## Data Availability

External researchers can make written requests for data sharing before publication or presentation. Requests will be assessed on a case-by-case basis in consultation with the lead and coinvestigators. A brief analysis plan and data request will be required and reviewed by the investigators to approve data sharing. In all cases, a data transfer agreement (DTA) will have to be signed through our Data Protection and Legal Office before any data can be shared. After signing the DTA, data will be sent electronically as password-protected files. All data sharing will abide by the rules and policies defined by the sponsor, relevant institutional review boards, and local, state and federal laws and regulations. The data sharing mechanisms will ensure that the rights and privacy of individuals participating in research sponsored by the NIH will be protected at all times.

## Code availability

External researchers can make written requests for the code used in the statistical analysis from R. Muwonge (muwonger@iarc.who.int). These requests should include a brief explanation of what the code is going to be used for. Each request will then be discussed with and approved by the lead and coinvestigators. Approval will be done within a month of receiving the request.
