# Supplementary material for: A portable thermal ablation device for cervical cancer prevention in a screen-and-treat setting: a randomized, noninferiority trial
Source: Nat Med. 2024 Jun 25;30(9):2596–604. doi: 10.1038/s41591-024-03080-w (PMC11405263; doi:10.1038/s41591-024-03080-w)
Supplement: Supplementary file 1 — Reporting Summary [file 41591_2024_3080_MOESM1_ESM.pdf]

Corresponding author(s): Dr. Partha Basu

Last updated by author(s): May 16, 2024

Reporting Summary

Nature Portfolio wishes to improve the reproducibility of the work that we publish. This form provides structure for consistency and transparency in reporting. For further information on Nature Portfolio policies, see our Editorial Policies and the Editorial Policy Checklist.

Statistics

For all statistical analyses, confirm that the following items are present in the figure legend, table legend, main text, or Methods section.

n/a	Confirmed	
☐	☒	The exact sample size (*n*) for each experimental group/condition, given as a discrete number and unit of measurement
☐	☒	A statement on whether measurements were taken from distinct samples or whether the same sample was measured repeatedly
☐	☒	The statistical test(s) used AND whether they are one- or two-sided<br>*Only common tests should be described solely by name; describe more complex techniques in the Methods section.*
☐	☒	A description of all covariates tested
☐	☒	A description of any assumptions or corrections, such as tests of normality and adjustment for multiple comparisons
☐	☒	A full description of the statistical parameters including central tendency (e.g. means) or other basic estimates (e.g. regression coefficient) AND variation (e.g. standard deviation) or associated estimates of uncertainty (e.g. confidence intervals)
☐	☒	For null hypothesis testing, the test statistic (e.g. *F*, *t*, *r*) with confidence intervals, effect sizes, degrees of freedom and *P* value noted<br>*Give P values as exact values whenever suitable.*
☒	☐	For Bayesian analysis, information on the choice of priors and Markov chain Monte Carlo settings
☒	☐	For hierarchical and complex designs, identification of the appropriate level for tests and full reporting of outcomes
☐	☒	Estimates of effect sizes (e.g. Cohen's *d*, Pearson's *r*), indicating how they were calculated

Our web collection on statistics for biologists contains articles on many of the points above.

Software and code

Policy information about availability of computer code

Data collection	Research Electronic Data Capture (REDCap 14.0.23) hosted at the International Agency for Research on Cancer (IARC)
Data analysis	STATA software, version 17.0 (Stata-Corp, College Station, TX, USA)

For manuscripts utilizing custom algorithms or software that are central to the research but not yet described in published literature, software must be made available to editors and reviewers. We strongly encourage code deposition in a community repository (e.g. GitHub). See the Nature Portfolio guidelines for submitting code & software for further information.

Data

Policy information about availability of data

All manuscripts must include a data availability statement. This statement should provide the following information, where applicable:

- Accession codes, unique identifiers, or web links for publicly available datasets
- A description of any restrictions on data availability
- For clinical datasets or third party data, please ensure that the statement adheres to our policy

Data Availability
External researchers can make written requests for sharing of data before publication or presentation. Requests will be assessed on a case by-case basis in consultation with lead and co-investigators. A brief analysis plan and data request will be required and reviewed by the investigators for approval of data sharing. In all cases, a data transfer agreement (DTA) will have to be signed through our Data Protection and Legal Office before any data can be shared. After signing the DTA,

data will be sent electronically in password protected files. All data sharing will abide by rules and policies defined by the sponsor; relevant institutional review boards; local, state, and federal laws and regulations. Data sharing mechanisms will ensure that the rights and privacy of individuals participating in research sponsored by the US National Institutes of Health will be protected at all times.

Code availability
External researchers can additionally make written requests for code used in the statistical analysis from Dr. Richard Muwonge (email: muwonger@iarc.who.int). These requests should include a brief explanation of what the code is going to be used for. Each request will be then be discussed with and approved by the lead and co-investigators. The approval will be done within a month of receipt of the request.

Research involving human participants, their data, or biological material

Policy information about studies with human participants or human data. See also policy information about sex, gender (identity/presentation), and sexual orientation and race, ethnicity and racism.

Reporting on sex and gender	We recruited adult female subjects only as the subject under consideration (cervical screening and treatment of precancers) is relevant to them only
Reporting on race, ethnicity, or other socially relevant groupings	The study was conducted in Zambia. All the recruited participants were black African women.
Population characteristics	Out of the total 3124 women recruited, 38.5% (N=1203) were aged 25 to 29, 39.8% (N=1244) were 30 to 39, 18.8% (N=587) were 40 to 49, and the rest (N=90; 2.9%) were aged 50 to 59 years (Table 1). The age distribution of participants belonging to cryotherapy and thermal ablation arms was evenly matched while in the LLETZ arm proportion of participants in the 25-29 year age group was comparatively less. Details of other demographic and reproductive factors are provided in table 1 and no significant difference was observed between the arms.<br>HIV status was known for 3071 participants, 58.6% (N=1801) being HIV positive. The majority (59.5%) of HIV positive women were known to have HIV infection for more than two years. Almost all HIV positive women were on ART at the time of recruitment. HIV positivity was 55.2% for women allocated to TA, 55.3% for cryotherapy, and 65.4% in the LLETZ arm (Table 1).
Recruitment	Screen eligible women attending primary health clinics (Chawama Level 1 Hospital, Chipata Level 1 Hospital, Kanyama Level 1 Hospital, Levy Mwanawasa Hospital, Matero Clinic) and University Teaching Hospital in Lusaka, Zambia comprised the target population for our study. Many of these women attend the clinics to receive medicines for anti-retroviral therapy (ART). All women within eligible age attending the clinics are routinely counselled to participate in the Zambian national cervical screening programme of the country. The clinics follow the Zambian national protocol that recommends women aged 25 to 49 years to be tested with visual inspection after application of acetic acid (VIA) and VIA-positive women be offered immediate treatment (screen and treat approach). Women aged up to 59 years who have never been screened before are also offered VIA.<br>Women willing to undergo screening are examined by a trained nurse at the clinic to perform VIA. VIA positive women are further assessed for eligibility for ablative treatment using the following criteria:<br>• The TZ is type I (entire squamo-columnar junction is visible at the external os or the ectocervix)<br>• The acetowhite area on the TZ does not occupy more than 75% of the ectocervix<br>• The acetowhite area does not extend to the endocervix or to the vagina<br>• There is no suspicion of invasive cervical cancer on visual examination<br>Women who were VIA positive and also eligible for ablative treatment were approached to participate in our study.<br>Selection and recruitment of study participants<br>VIA-positive women who fulfilled the criteria for suitability for ablative treatment and showed interest to participate in the study were further assessed by a trained social worker for following inclusion and exclusion criteria before she could initiate informed consenting process:<br>Inclusion criteria<br>• Age between 25 and 59 years<br>• VIA test positive<br>• Eligible for ablative treatment based on criteria described above; additionally, the TZ could be covered by a single application of largest cryotherapy probe<br>Exclusion criteria<br>• Pregnant at the time of recruitment<br>• Not in a position to provide voluntary informed consent due to mental illness or other medical conditions<br>During consenting process, the social worker explained the objectives, treatment procedures, follow up requirements, benefits and harms of participating in the study and responded to any queries. Each woman voluntarily willing to participate signed a written consent form before her recruitment to the study was finalized.<br>Due to the nature of recruitment self-selection bias and healthy volunteer effect can not be ruled out
Ethics oversight	The study was reviewed and approved by the research ethics committees at IARC and UNC. The collaborators at University Teaching Hospital, Zambia had major roles in study design, study implementation, data analysis and manuscript preparation. A DSMB was constituted with five members including a lay person to monitor the study from time to time. Study was registered at the clinical trials registry (ClinicalTrials.gov: DELTA study NCT02956239).

Note that full information on the approval of the study protocol must also be provided in the manuscript.

Field-specific reporting

Please select the one below that is the best fit for your research. If you are not sure, read the appropriate sections before making your selection.

☒ Life sciences ☐ Behavioural & social sciences ☐ Ecological, evolutionary & environmental sciences

For a reference copy of the document with all sections, see nature.com/documents/nr-reporting-summary-flat.pdf

Life sciences study design

All studies must disclose on these points even when the disclosure is negative.

Sample size	In the pilot phase of the study 250 women were empirically allocated to either of the treatment arms. Sample size was calculated prior to the extended phase using data from the pilot. To demonstrate non inferiority in the treatment success rates, we used the following assumptions:<br>• Treatment success rate in cryotherapy (comparator) arm to be 55% [Rationale: treatment success rate in the cryotherapy arm at 6 months follow-up at the end of the pilot phase was 55%. We use this as a conservative treatment success rate at 12 months]<br>• Difference in treatment success rates between thermal ablation and cryotherapy to be 5% [Rationale: At the end of the pilot UH2 phase difference of 5% in treatment success rates at 6-month follow-up was observed (60% treatment success rate in the thermal ablation arm)]<br>• A non-inferiority margin of 4%.<br>• A follow-up annual default rate of 30%;<br>• An 80% power; and<br>• A 2.5% level of significance.<br>• In order to account for the three-arm multiple comparisons and for the planned sub-analyses stratified by HIV status, a 0.00417 (0.025/3/2) level of significance was used in the calculations<br>It was estimated that a total sample size of 3,123 VIA screen positive women would be required to confidently answer the primary questions posed in this study. This was to be equally randomized to the 3 arms making 998 women per arm. The pilot study recruited total 750 participants. Based on the formal sample size estimation, additional 2,373 participants were planned to be recruited in the extended phase of the RCT.
Data exclusions	No data was excluded
Replication	Not applicable
Randomization	The recruited women were randomly assigned to any of the three treatment groups in 1:1:1 ratio. Before the launch of the study, a randomization list was generated by IARC data manager using random function in excel to associate a treatment to each participant ID. This list was not shared with investigators in Zambia. An IARC study coordinator inserted treatment allocations printed on the participant barcode sheets with a specific color code in opaque sealed envelopes with only the randomization sequence written on the envelopes. These envelopes were mailed to the site coordinator in Zambia who arranged them serially by the sequence number. The social worker called the site coordinator over telephone after confirming participation of an eligible woman to know the treatment allocation. The social worker conveyed treatment allocation to the treating nurse. Once allocated to a particular treatment arm the treatment modality could not be changed.
Blinding	Blinding of either the participant or the treating nurse was not feasible due to the nature of interventions because the three treatments use different devices. The assessors (nurses performing follow up procedures and the lab persons analysing the HPV tests at follow up) were blinded

Reporting for specific materials, systems and methods

We require information from authors about some types of materials, experimental systems and methods used in many studies. Here, indicate whether each material, system or method listed is relevant to your study. If you are not sure if a list item applies to your research, read the appropriate section before selecting a response.

Materials & experimental systems

n/a	Involved in the study	
☒	☐	Antibodies
☒	☐	Eukaryotic cell lines
☒	☐	Palaeontology and archaeology
☒	☐	Animals and other organisms
☐	☒	Clinical data
☒	☐	Dual use research of concern
☒	☐	Plants

Methods

n/a	Involved in the study	
☒	☐	ChIP-seq
☒	☐	Flow cytometry
☒	☐	MRI-based neuroimaging

Clinical data

Policy information about clinical studies

All manuscripts should comply with the ICMJE guidelines for publication of clinical research and a completed CONSORT checklist must be included with all submissions.

Clinical trial registration	Registered with ClinicalTrials.gov (number NCT02956239)
Study protocol	The description of the study protocol is provided in the clinical trial registration and may be directly request from the corresponding author
Data collection	Screen eligible women attending primary health clinics (Chawama Level 1 Hospital, Chipata Level 1 Hospital, Kanyama Level 1 Hospital, Levy Mwanawasa Hospital, Matero Clinic) and University Teaching Hospital in Lusaka, Zambia. The first participant was recruited on August 2, 2017, and the last one on September 29, 2022.
Outcomes	The primary outcome of interest was treatment success. As per a pre-specified analysis plan success of treatment at 12-month follow up at 12 months was defined as either HPV type-specific clearance among participants positive for high-risk HPV at baseline, or negative VIA test if the baseline HPV test was negative. The secondary outcomes were treatment side effects and complications, and patient reported outcomes (pain and discomfort and level of satisfaction) immediate post-treatment and two weeks later. Effect estimates were provided as relative risks (RRs) together with their 95% confidence intervals (CIs) obtained from the generalized linear models for the binomial family with the log link function. The secondary outcomes were shown as proportions, with the comparison between the three study arms was carried out using the Pearson chi-square test.
